# P-954. Integration Station - The Train Stops Here: Measuring Effectiveness of Integration of Medicinal Chemistry and Clinical Practice Content in a Required Infectious Diseases Pharmacotherapy Course for Pharmacy Students

**DOI:** 10.1093/ofid/ofae631.1144

**Published:** 2025-01-29

**Authors:** Marcelo Nieto, Beth Cady

**Affiliations:** Southern Illinois University at Edwardsville School of Pharmacy, Edwardsville, Illinois; Southern Illinois University at Edwardsville School of Pharmacy, Edwardsville, Illinois

## Abstract

**Background:**

Pharmacy school accrediting bodies require curricular integration with medicinal chemistry, pharmacology and practice in the curriculum. No studies discuss how to execute that integration effectively. Two pharmacy faculty (one from pharmaceutical sciences and one from pharmacy practice) developed an innovative method to integrate their content in a required ID course. The goal was to determine if exam scores improved.

**Methods:**

Two faculty at one school of pharmacy developed an innovative method to deliver integrated content in a required ID pharmacotherapy course. They utilized methods of backward design and in-class application during the 2024 course delivery. Objectively, exam scores were compared to prior years (both overall scores and individual content questions such as pharmaceutical science and pharmacy practice) to assess effectiveness of content delivery.

**Results:**

All of the infectious diseases content was assessed on two exams (exam 3 and 4) in the required course. The exam 3 average from 2024 was 71% and average scores from the three years prior were 72% (2023), 73.3% (2022), and 65.8% (2021). The exam 4 average from 2024 was 80.4%, and past average scores were 68.4% (2023), 69.3% (2022), and 67.1% (2021). Individual content between pharmaceutical sciences and pharmacy practice content from the exam scores from 2024 were higher than all previous exam scores, with a range of 1.6-14% points better in practice content, and 1.6-12.2% points better in science content.

**Conclusion:**

While there may not have been a dramatic increase in exam scores from the 2024 iteration compared to the previous years, it can be noted that there were some improvements with the newly implemented method of integrated content delivery. Despite the fact that the overall exam 3 average did not increase in 2024 compared to 2023/2022, it is important to note that the content of the exam was weighted differently. There was 1.5x the amount of medicinal chemistry content on the 2024 exam vs previous exams. Students tend to perform worse on medicinal chemistry content. With improved exam scores, this method of integrated science and practice content appears to have been delivered more effectively than in past years.

**Disclosures:**

**All Authors**: No reported disclosures

